# Epidemiology and Incidence of Upper Limb Fractures: A UK Level 1 Trauma Center Perspective

**DOI:** 10.7759/cureus.54961

**Published:** 2024-02-26

**Authors:** James Zhang, Florence Bradshaw, Michal Duchniewicz, Ioannis Karamatzanis, Fernanda W Fernandes, Matjia Krkovic

**Affiliations:** 1 School of Clinical Medicine, University of Cambridge, Cambridge, GBR; 2 Trauma and Orthopedics, Addenbrooke's Hospital, Cambridge University Hospitals NHS Foundation Trust, Cambridge, GBR; 3 Internal Medicine and General Surgery, Basildon University Hospital, Basildon, GBR; 4 Surgery, University College Hospital, London, GBR

**Keywords:** epidemiology, incidence, phalanges, radius, demographic, fracture, trauma, ulna, humerus

## Abstract

Introduction

Understanding the incidence and epidemiology can inform clinicians and policymakers about the population's needs. Our study reports on upper limb fractures treated at a major trauma center over 7.5 years.

Methods

We collected data on fracture locations, age, gender, Charlson Comorbidity index (CCI), and treatment options of all upper limb fractures treated at a Level I Trauma Centre from January 1, 2015 to June 30, 2022. Humerus, radius, and ulna fractures were each classified as proximal, diaphyseal, and distal.

Results

About 9,915 patients sustained 12,790 fractures, given an overall incidence of 303.2 fractures per 100,000 patients per year. The most common fracture site was the distal radius (60.1 fractures per 100,000 patients per year, whereas carpal and metacarpal bones had the lowest incidence. The mean age and CCI were 46.4 years and 1.54, respectively. 58.1% of patients were male. All bone fractures distal to the elbow were associated with an age younger than the mean (all p<0.001), with humerus fracture patients having the oldest mean age (54.6 years). Compared to the mean gender ratio, except for ulna (no association), humerus (55% female), and radius (51% female), all other locations showed significantly higher incidences of males (all p<0.001). When plotting the incidence based on the age of injury, the entire cohort, along with radius and ulna fracture subgroups, demonstrated a bi-peak distribution. This pattern revealed that younger males and older postmenopausal females had the highest incidence rates.

Conclusion

To our knowledge, this represents the first study of this type in the UK since 2006. We sought to elucidate relative incidence and demographic associations with fractures to highlight changing population needs and allow policymakers and services at a regional and national level to operate with up-to-date information.

## Introduction

Understanding the demographics, patterns, and underlying trends in fractures in the population allows for a better understanding of the current and evolving needs of the population. There has yet to be a recent, large UK-based study on the epidemiology of upper limb fractures. The latest research paper that delved into the epidemiology of upper limb fractures in the UK dates back to 2006 [1]; however, this paper also examined fractures in other locations. A more recent study in 2015 explored fracture epidemiology in the UK, but it categorized fractures based on generalized body areas rather than specific upper limb fractures [2]. Notably, both papers only covered a one-year time span. Moreover, a significant disparity exists in the reported incidence of fractures in the UK, ranging from 98 to 132.7 per 10,000 population [3]. The underlying reasons for this considerable fluctuation still need to be determined, although they may stem from variations in data sources. For instance, orthopedic fracture clinic data frequently fails to capture all fractures since not all fractures necessitate referral there. Conversely, data from Accident and Emergency departments might lead to overestimating fracture incidence due to potential over-diagnosis of certain types of fractures [1]. Additionally, the methodology employed to calculate the overall population for determining incidence rates contributes to the observed variations in fracture incidence.

Analyzing data that delineates the epidemiological patterns of fractures holds significant importance, as it provides valuable insights to clinicians and policymakers regarding the population's requirements. This, in turn, aids in the strategic planning of healthcare delivery. Such an analysis can also unveil which genders are susceptible to specific fractures, along with correlations involving age, BMI, and comorbidities in relation to fracture patterns. Notably, the demographic analysis aspect gains heightened significance within aging populations, as seen in the case of the UK, where it helps pinpoint fractures that could be categorized as osteoporotic. It is noteworthy that in 2019, osteoporotic fractures accounted for approximately 2.4% of healthcare expenditures in the UK [4]. The identification of osteoporotic fractures and individuals with an elevated risk of fracture could play a pivotal role in fracture prevention, subsequently leading to a reduction in the overall expenses associated with osteoporotic fractures within the UK healthcare budget.

To comprehensively define the epidemiology of upper limb fractures, we conducted a retrospective study encompassing a 7.5-year timeframe, focusing on upper limb fractures treated at a Major Trauma Center within the UK.

## Materials and methods

Situated in the Cambridgeshire region, Addenbrooke's Hospital is a Major Trauma Centre catering to a population of around 570,000 patients. This study used the Epic Systems^TM^ database to identify patients who visited Accident and Emergency (A&E) and orthopedic outpatient departments. Inclusion criteria encompassed patients with specific upper limb fracture diagnoses, as indicated by ICD-10 clinical coding.

The study's timeline extended over seven years, from January 1, 2015 to June 30, 2022. All fractures located distal to and including the scapula and clavicle were included. Furthermore, each fracture category underwent a subsequent classification based on whether it was an open or closed fracture type. The scope of included bones comprised the clavicle, scapula, humerus, ulna, radius, carpal, metacarpal, and phalanges. The humerus, ulna, and radius were subjected to further subdivision, and categorized into their proximal, shaft, and distal segments. Instances, where multiple fractures of varying bone types occurred within the same patient and admission, were treated as distinct occurrences.

Demographic features such as age at admission, Body Mass Index (BMI), gender, ASA grade (for surgically managed patients), and Charlson Comorbidity Index (CCI) score were calculated for each patient. The study then analyzed the demographic breakdown for each fracture sublocation and type. Graphs were generated to display the frequency of fractures for each bone location in relation to patient age, and separate graphs for each gender to identify trends in the fracture data. Additionally, the quarterly frequency of fractures over the seven-and-a-half-year period was calculated and graphed, along with location subtype graphs, to examine the impact of the COVID-19 pandemic and subsequent lockdowns on acute upper limb injuries. Furthermore, an in-depth analysis was conducted to investigate potential differences in patient demographics and the percentage of surgically managed cases over time. A one-way ANOVA (analysis of variance) was performed, treating each year (2015-2022) as a separate group. This statistical test allows for comparisons between multiple groups simultaneously, similar to a T-test comparing two groups. A significance level of p<0.05 was considered statistically significant.

Variables such as the percentage of surgically managed cases, patient age, gender, ASA grade, BMI, and Charleston Comorbidity Index were compared across the years. If a variable showed significant differences (p<0.05), post-hoc analysis using the Bonferroni post-hoc test with Bonferroni correction was conducted to identify specific significant comparisons. This test ensures that when there are multiple T-tests performed across an array of data groups, the p-value threshold is accordingly adjusted and reduced, to ensure the increase in type I error inherent to multiple statistical tests is offset. The manuscript includes means for each investigated category in each year to provide context for significant findings, ANOVA significance levels, and post-hoc test results displayed in tables. Additional supplementary tables present mean differences, standard deviations/errors, and confidence intervals. Data analysis and graph construction were performed using SPSS v28 (IBM Corp., Armonk, NY) and Microsoft Excel^TM^.

## Results

A total of 12,790 fractures were recorded from 9,915 patients over the study's time frame. In our study, the average age was 48.2 years, with 56.4% of patients being male. The overall incidence of upper limb fractures was 303.2 fractures per 100,000 patients per year. This value is considerably lower than quoted in previous studies [1] and is discussed in the limitations.

Location

The breakdown by location and open vs. closed nature of fractures are displayed in Table 1. The most common fracture site was the distal radius, with 20.1% of all fractures being of that location, representing an incidence of 60.1 distal radial fractures per 100,000 patients per year. Carpal and metacarpal bones were of lowest incidence, with the humerus and ulna being roughly similar in incidence, at 18.6% and 18.8%, respectively, when combining fractures occurring proximally, at the shaft, and distally. Phalangeal fractures were the most likely to be open injuries, with 50.7% and 59.8% of thumb and other phalangeal fractures presenting in this fashion, whereas only 1.38% of clavicular fractures were open. Overall, 17.5% of all fractures were open.

**Table 1:** Breakdown of fractures by location and open vs. closed nature

Location	Open vs Closed	Number	Location Percentage (%)	Location Incidence (/100,000 patients per year)	Percentage Open (%)
Clavicle	Closed	1219	9.6	28.9	1.38
	Open	17			
Scapula	Closed	633	5.1	15.1	2.01
	Open	13			
Proximal Humerus	Closed	1106	8.9	26.7	3.07
	Open	35			
Shaft of Humerus	Closed	300	2.8	8.4	16.90
	Open	61			
Distal Humerus	Closed	729	6.8	20.2	15.63
	Open	135			
Proximal Radius	Closed	398	3.4	10.2	8.92
	Open	39			
Shaft of Radius	Closed	421	4.2	12.5	21.01
	Open	112			
Distal Radius	Closed	2311	20.1	60.1	10.11
	Open	260			
Proximal Ulna	Closed	733	7.1	21.4	19.80
	Open	181			
Shaft of Ulna	Closed	438	4.3	13.1	21.51
	Open	120			
Distal Ulna	Closed	769	7.4	22.1	18.71
	Open	177			
Scaphoid	Closed	208	1.7	5.1	4.59
	Open	10			
Other Carpal Bone(s)	Closed	133	1.3	3.8	18.40
	Open	30			
First Metacarpal	Closed	124	1.1	3.2	8.82
	Open	12			
Other Metacarpal	Closed	327	3	9.1	15.50
	Open	60			
Thumb Phalanges	Closed	142	2.2	6.7	50.69
	Open	146			
Other Phalanges	Closed	559	10.8	32.5	59.81
	Open	832			

Demographic breakdown 

The demographic breakdown of each fracture location and subtype is displayed in Table 2. The oldest average age of fracture by location was proximal humeral (69.83 years old), with the youngest average age for the radial shaft (24.42 years old) followed by the ulnar shaft (26.51 years old). Most locations showed a higher incidence among males, with the first metacarpal being the most skewed (84% male), whereas the proximal humerus had the highest incidence of females (63% female). The BMI measurements across different locations showed very little overall trend in this population.

**Table 2:** Further demographic breakdown of each fracture location and subtype. For P-values and standard deviations, see Supplementary Table A.

Fracture Location	Number	Age Mean	Proportion Female	ASA Grade Mean	BMI Mean	% Surgically managed	CCI Mean
Clavicle	1236	50.86	0.33	2.25	25.32	27.62	1.71
Scapula	646	53.27	0.26	3.00	26.13	21.06	1.82
Proximal Humerus	1141	69.83	0.63	2.57	25.16	18.31	3.39
Shaft of Humerus	361	52.48	0.43	2.85	25.91	45.39	1.99
Distal Humerus	864	34.59	0.49	1.92	23.26	39.80	1.14
Proximal Radius	437	47.08	0.46	2.14	25.89	37.27	1.41
Shaft of Radius	533	24.42	0.32	1.78	23.06	61.36	0.30
Distal Radius	2571	49.16	0.54	1.93	25.13	35.66	1.76
Proximal Ulna	914	48.48	0.47	2.10	25.49	39.13	1.44
Shaft of Ulna	558	26.51	0.35	1.84	23.60	62.02	0.40
Distal Ulna	946	41.69	0.46	2.01	24.35	38.30	1.35
Scaphoid	218	42.09	0.29	1.77	25.62	20.69	1.22
Other Carpal Bone(s)	163	43.69	0.21	2.45	26.39	47.23	1.12
First Metacarpal	136	37.17	0.16	1.43	26.81	12.50	0.72
Other Metacarpal	387	46.21	0.24	2.11	25.91	25.09	1.30
Thumb Phalanges	288	46.72	0.27	1.78	25.66	6.60	1.57
Other Phalanges	1391	41.59	0.27	1.60	25.43	5.17	0.97

ASA grade, surgical management 

During the study, 3,993 surgeries were performed on 12790 fractures. Surgeries included external fixation, intramedullary nails, or plate fixation. The shaft of the ulna and radius had the highest number of operatively treated fractures, while metacarpal and phalangeal fractures had the lowest surgical rates. 

Charlson comorbidity index

Finally, we calculated each patient's CCI. We found that patients with fractures of the shaft of the ulna and radius showed the lowest CCI means, indicating younger and more active patients sustaining these location fractures. In contrast, patients with proximal humerus fractures were the most frail (highest CCI).

Age, gender, and fracture patterns 

**Figure 1:**
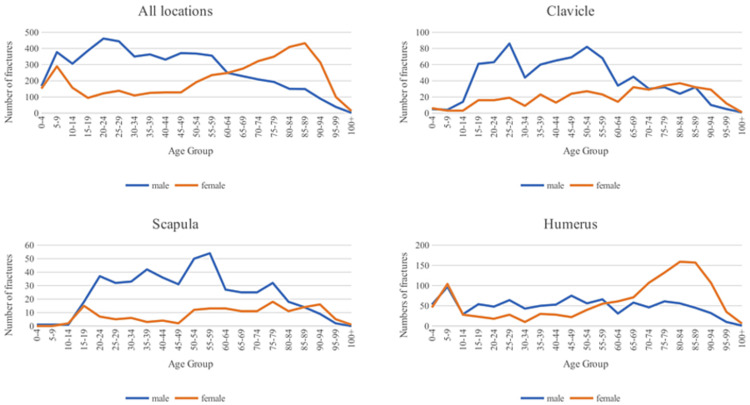
Fracture locations incidence by gender graphed with respect to age

**Figure 2:**
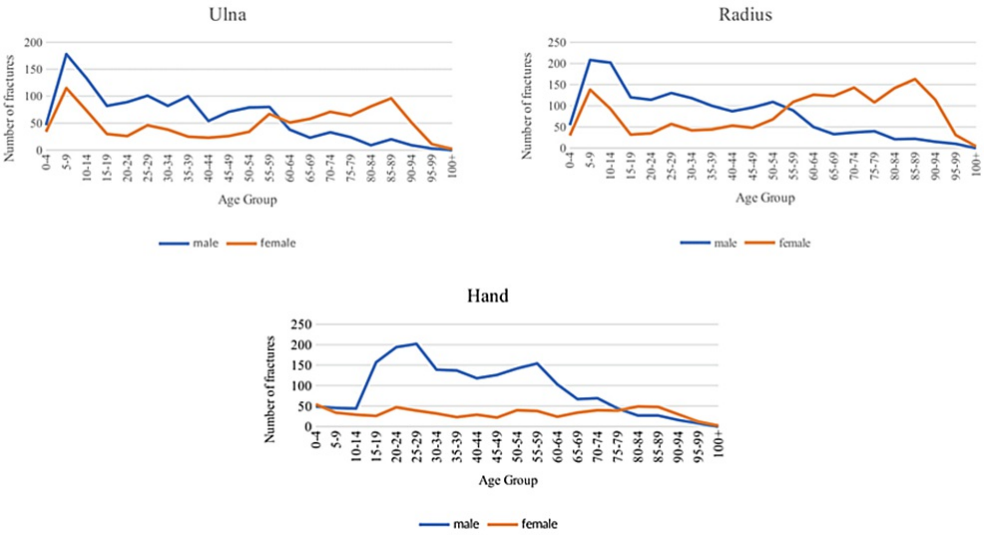
Forearm fracture locations incidence by gender graphed with respect to age

Fracture locations and the entire cohort were graphed by age and gender in Figures 1, 2, following the methods introduced by Burh et al. in 1959 and later utilized by Court-Brown et al. to characterize fracture patterns [1,5]. The overall graph and humerus, ulna, and radius fractures exhibit a pattern where younger males and older females have gender-specific peaks.

Upper limb fractures over time

**Figure 3:**
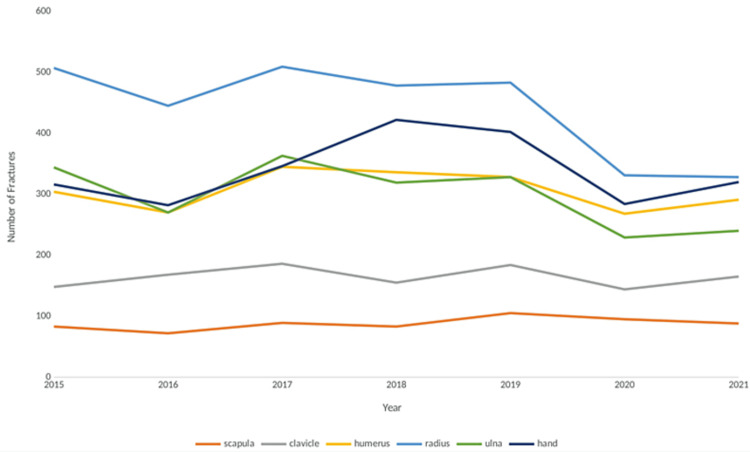
Incidence of fractures over the seven-year time period, stratified by location

**Figure 4:**
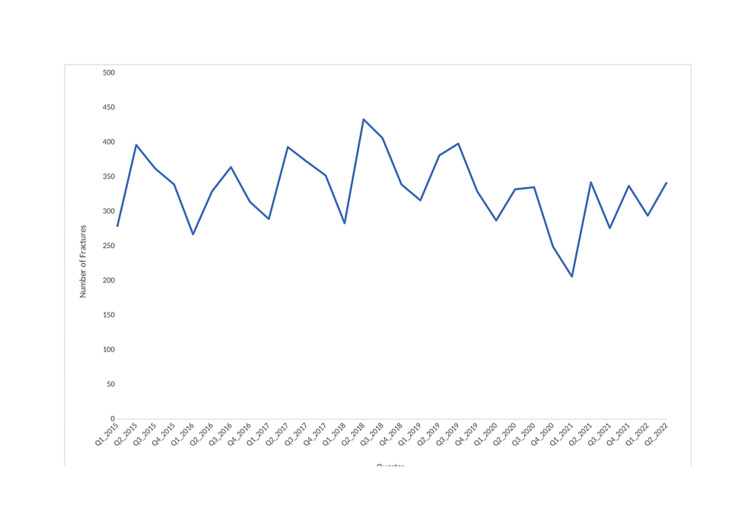
Incidence of fractures by quarter over the seven-year time period

The incidence of each fracture sub-location by year is shown in Figure 3, and the overall incidence by quarter is shown in Figure 4.

From 2015 to 2019, upper limb fractures remained relatively constant, averaging 1,392 per year. However, in 2020 and 2021, lower numbers of fractures were recorded (1,067 and 1,151 fractures, respectively). This trend was seen in ulna and radius fractures, but a less drastic decrease occurred for most other locations. In 2020 and 2021, there was a decrease in the number of upper limb fractures in the fourth quarter of 2020 and the first quarter of 2021. When splitting each year into quarters, the number of fractures oscillates, with increases in numbers generally seen in the second and third quarters of each year. The one-way ANOVA test for % fractures surgically managed, age, gender, ASA grade, BMI, and CCI score, alongside each year's mean, are shown in Table 3.

**Table 3:** One-way ANOVA analysis of various demographic and management factors across each year included in the study *Significance at p<0.05

Factor	One Way ANOVA significance (p-value)	2015	2016	2017	2018	2019	2020	2021	2022
Management (% surgically managed)	0.157	0.55	0.55	0.56	0.55	0.53	0.55	0.53	0.60
Age (years)	0.003*	46.37	47.63	47.63	47.69	48.04	50.50	49.95	49.58
Gender (%female)	0.264	0.46	0.42	0.44	0.42	0.44	0.42	0.45	0.45
ASA grade	<0.001*	1.76	1.77	1.91	1.86	1.94	1.98	2.01	1.89
BMI	0.277	24.89	25.15	25.16	25.03	24.76	25.34	25.11	25.53
CCI score	<0.001*	1.55	1.68	1.61	1.64	1.78	1.96	1.93	1.78

Only the factors of age, ASA grade, and CCI score reached significance in this model. This highlights that there were no significant changes in the percentage of fractures surgically managed, gender, and BMI of patients presenting with upper limb fractures over this time period. Subsequently, post-hoc analysis with the Bonferroni method was conducted for age, ASA grade, and CCI score, shown in Table 4.

**Table 4:** Post-hoc analysis with the Bonferroni method was conducted for age, ASA grade, and CCI score *Significance at p<0.05. Please see Supplementary Table C for further breakdown of the Bonferroni method results.

Factor	Reference/Comparison year								
Age		2015	2016	2017	2018	2019	2020	2021	2022
	2015		1.0000	1.0000	1.0000	1.0000	0.0058*	0.0284*	0.2792
	2016	-		1.0000	1.0000	1.0000	0.3328	1.0000	1.0000
	2017	-	-		1.0000	1.0000	0.2801	0.9357	1.0000
	2018	-	-	-		1.0000	0.3076	1.0000	1.0000
	2019	-	-	-	-		0.7509	1.0000	1.0000
	2020	-	-	-	-	-		1.0000	1.0000
	2021	-	-	-	-	-	-		1.0000
	2022	-	-	-	-	-	-	-	
ASA grade									
	2015		1.0000	0.1403	1.0000	0.0218*	0.0024*	0.0003*	0.9520
	2016	-		0.2700	1.0000	0.0489*	0.0060*	0.0008*	1.0000
	2017	-	-		1.0000	1.0000	1.0000	1.0000	1.0000
	2018	-	-	-		1.0000	0.7928	0.2082	1.0000
	2019	-	-	-	-		1.0000	1.0000	1.0000
	2020	-	-	-	-	-		1.0000	1.0000
	2021	-	-	-	-	-	-		1.0000
	2022	-	-	-	-	-	-	-	
CCI score									
	2015		1.0000	1.0000	1.0000	0.2008	0.0003*	0.0009*	0.8577
	2016	-		1.0000	1.0000	1.0000	0.1047	0.2220	1.0000
	2017	-	-		1.0000	1.0000	0.0045*	0.0111*	1.0000
	2018	-	-	-		1.0000	0.0148*	0.0349*	1.0000
	2019			-	-		1.0000	1.0000	1.0000
	2020	-	-	-	-	-			1.0000
	2021	-	-	-	-	-	-		1.0000
	2022	-	-	-	-	-	-	-	

For ages, 2020 and 2021, patients had significantly higher ages compared to 2015. For ASA grades, 2019, 2020, and 2021 patients had higher grades compared to 2015 and 2016 patients. For CCI score, 2020 and 2021 patients had greater comorbidities compared to 2015, 2017, and 2018.

## Discussion

Our study investigated all upper limb fractures presenting at a major trauma center over the span of seven years. We sought to elucidate demographic trends and patterns, sub-location breakdown insights, and temporal differences. The study showed that the distal radius was the most common site for fractures; however, the shaft of the ulna and radius had the highest number of operatively treated fractures. A 2006 paper by Court-Brown et al. noted that distal radial fractures were the most prevalent, whereas scapula fractures had the lowest incidence [1]. Similarly, a study conducted in 2009 in the US found radial fractures to be the most frequent, while clavicle fractures were the least common [6]. These large-scale epidemiology studies indicate a consensus on the most prevalent upper limb fracture. However, the least common upper limb fracture remains to have a concurrence. Fractures of the radius and ulna are often the result of high-energy trauma, such as road traffic accidents in young individuals and adults. Conversely, low-energy trauma is a common cause of forearm fractures, usually attributed to compromised bone quality. Pediatric forearm fractures seldom require surgical intervention, unlike adults who frequently need reduction and immobilization prior to surgery to improve patient outcomes [7]. These observations support the high surgery rates for radius and ulna shaft fractures.

The American Society of Anesthesiologists (ASA) physical status grading predicts operative risk by considering patients' comorbidities [8]. Generally, higher ASA grades were associated with proximal fractures at presentation or during the initial operation, with the scapula having the highest average grade of 3.00. While limited literature directly links ASA grade to fracture location, it is worth noting that ASA grade can independently forecast the likelihood of readmission after a fracture [9].

Demographic breakdown of upper limb fractures

Body Mass Index

Our BMI measurements across different locations showed very little overall trend in this population. However, obesity is a growing issue in the UK, with estimates projecting that the percentage of the population in England who are morbidly obese (BMI > 40) will reach 8% in 2035 [10]. A previous study found a positive relationship between obesity and proximal humerus fractures in males and a positive relationship between BMI and shaft of humerus fractures in females. A negative relationship was found between BMI and clavicle fractures in both genders. However, the paper concludes that there are likely to be many factors involved in fracture epidemiology, of which BMI is a minor factor [11]. This sentiment is echoed in a study from 2020, showing that there is an increased incidence of humeral fractures in obese patients compared to those of the wrist in the same patient group [12]. 

*Charlson Comorbidity Index*

Proximal humeral occurred in the eldest age demographic with the highest CCI means, with the radial and ulnar shaft most commonly occurring in the third decade of life for patients. Despite this, CCI was shown in a previous study to be a poor predictor in calculating the risk of hip fractures and osteoporotic fractures. A new Charlson Fracture Index (CFI) was created to predict hip fractures in men and women. This tool could identify patients at an increased risk of hip fracture [13]. It would be intriguing to explore whether the CFI could serve as a predictive measure for upper limb fractures, potentially forming the basis for future research endeavors. Despite earlier indications that CCI is not a robust predictor for calculating fracture vulnerability, it is important to highlight that CCI has demonstrated efficacy as a predictor of mortality, particularly in cases of proximal humerus fractures [14].

Age, Gender, and Upper Limb Fracture Patterns

The overall graph and graphs for the humerus, ulna, and radius fractures show gender-specific peaks for younger males and older females. The rise in the incidence of fractures in females occurs around menopause, reflecting the increased fracture risk due to estrogen deficiency [15]. The peak incidence of fractures in younger males aligns with Court-Brown et al.'s findings, although they also note a gradual increase after age 60 [1]. This initial peak in males likely stems from younger men engaging in more physical risks and activities, leading to a higher likelihood of experiencing high-energy trauma [16]. Similarly, clavicle, scapula, and hand fractures, graphed by age, display distinct shapes within each gender, but the relative incidence between genders differs significantly. Males have a much higher incidence across various age ranges, with a crossover occurring around the 80-90 years old range. This discrepancy may be attributed to the scapula, clavicle, and hand fractures often resulting from traumatic injuries rather than fragility and osteoporosis, unlike humeral, ulnar, and radial fractures.

Fractures and Osteoporosis

The UK has an aging population, and osteoporotic fractures account for 2.4% of UK healthcare spending [4]. Osteoporosis is characterized by “compromised bone strength predisposing a person to an increased risk of fracture” [17]. On a cellular level, it can be considered a metabolic bone disease resulting from an imbalance between osteoclastic bone resorption and osteoblastic bone formation, leaving bones weak and fragile, thus increasing the risk of fracture. Factors contributing to the development of osteoporosis include estrogen or vitamin D deficiency, secondary hyperparathyroidism, and even the gut microbiome [17]. Traditional osteoporotic fractures were initially thought to be fractures of the thoracolumbar vertebrae, distal radius, proximal femur, and proximal humerus [1]. This helps to explain the graph for radial and humerus fractures. As the shape and relative trends of the ulna fractures are similar to that of the radius and humerus, this could suggest that previously unidentified osteoporotic fractures were responsible. In the future, as the UK population demographics age further, more fractures may be defined as osteoporotic, which will have implications for the prevention, detection, treatment, and management of these osteoporotic fractures. 

Upper Limb Fractures Over Time

From 2015 to 2019, upper limb fractures remained relatively constant, however, in 2020 and 2021, fewer fractures were recorded, particularly for ulna and radius fractures. This decrease coincided with the second and third UK COVID lockdowns, November to December 2020 and January to February 2021 [18]. Lim et al. reported a similar decrease in fractures but observed an increase in the proportion of hand fractures, which our study did not [19]. When splitting each year into quarters, the number of fractures oscillates, with increases in numbers generally seen in the second and third quarters of each year. This pattern could be driven by the summer months. MacDermid et al. found an increase in clavicle, radial head, and other fractures in regions of the upper extremity during the summer months, possibly due to increased participation in outdoor sports and recreational activities due to the warmer weather [20].

Only age, ASA grade, and CCI score reached significance in the ANOVA model. This highlights that there were no significant changes in the percentage of fractures surgically managed, gender, and BMI of patients presenting with upper limb fractures over this time period. The post-hoc analysis using the Bonferroni method showed that 2020 and 2021 patients had significantly higher ages, grades, and comorbidities than previous years. This indicates some limited evidence to support our previous results that over the two pandemic and lockdown-affected years, the patients presenting with fractures were more likely to be older and frailer due to the overall decrease in activity amongst the younger population; however, the lack of widespread statistical significance means this could nonetheless be a spurious finding. Our negative findings of no significant changes over time for surgical management percentage, gender, and BMI also contribute to the literature and give insight into the population investigated.

Limitations and further directions

Our overall incidence of upper limb fractures was considerably lower than reported in previous studies [1]. This could be attributed to several factors, mainly the uncertainty surrounding the population served by the trauma center. Although we examined the catchment area of the Level 1 Major Trauma Center, the incidence of fractures reported in this study may be influenced by individuals' decisions on which local A&E to visit and complex ambulance dispatch areas and routes. This limitation was also acknowledged in the 2006 study by Court-Brown et al. A comprehensive fracture epidemiology study covering the entire UK would be necessary to determine the true incidence. However, this would be a highly challenging endeavor, requiring coordination among multiple trusts across the country, but it could provide insights into potential regional differences in fracture incidence.​​​​​​ Another factor contributing to our lower reported fracture incidence is the recording of multiple fractures on the same bone and side of the body as a single fracture. For instance, multiple metacarpal fractures or a comminuted proximal humerus fracture would each be counted as a single fracture.

Furthermore, we did not utilize the AO OTA classification, which is commonly used in research but not in clinical practice. Instead, we relied on ICD-10 coding at the time of admission. However, it is worth noting that most epidemiology fracture papers also do not employ the AO OTA fracture classification. We anticipate readers will utilize our results to inform policy decisions and gain insights into population needs and epidemiology rather than conducting further research on fracture types and outcome associations.

A systematic review or meta-analysis examining fracture incidence across different countries would be beneficial to identify additional factors that could contribute to variations in fracture incidence for the same types of fractures. Climate, study methodologies, healthcare structures, and the likelihood of patients seeking emergency care for minor injuries are all potential factors that may impact reported fracture incidence.

## Conclusions

Our study sought to report upon, define and analyze the epidemiology of upper limb fractures in the United Kingdom. We have presented a large cohort, in a tertiary trauma center, and the subsequent detailed exploration of trends and associations. Finally, this has elucidated up-to-date information that equips clinicians and policymakers with the tools to meet and tailor healthcare services to the needs of a dynamic population, fostering more effective and responsive healthcare practices.

## Appendices

**Table 5:** Extended to include P-values and standard deviations for the further demographic breakdown of each fracture location and subtype N.B. Table split in two for formatting and ease of reading, the first two columns are repeated.

Fracture location	Number	Age mean	Age Standard Deviation	P-value	Proportion Female	P-value	ASA mean	ASA Standard Deviation	P value
Clavicle	1236	50.86	22.03	<0.001	0.33	<0.001	2.25	1.16	<0.001
Scapula	646	53.25	23.34	<0.001	0.26	<0.001	3	1.14	<0.001
Proximal Humerus	1141	69.84	19.83	<0.001	0.63	<0.001	2.57	0.98	<0.001
Shaft of Humerus	361	52.48	25.23	<0.001	0.43	0.638	2.85	1.14	<0.001
Distal humerus	864	34.6	29.04	<0.001	0.49	<0.001	1.92	1.06	<0.001
Proximal Radius	437	47.08	24.76	0.597	0.46	0.09	2.14	1.03	0.358
Shaft of radius	533	24.23	19.83	<0.001	0.33	<0.001	1.78	1.05	<0.001
Distal radius	2571	49.19	27.89	<0.001	0.54	0.002	1.93	1.01	<0.001
Proximal ulna	914	48.48	24.41	0.024	0.47	0.002	2.1	1.01	0.324
Shaft of ulna	558	26.36	21.26	<0.001	0.35	0.014	1.84	1.07	<0.001
Distal ulna	946	41.74	29.57	<0.001	0.46	<0.001	2.01	1.12	0.414
Carpal bone(s)	381	42.77	22.75	0.011	0.25	<0.001	2.06	0.99	0.454
Metacarpal(s)	523	43.61	23.37	0.023	0.22	<0.001	2.05	1.12	0.324
Phalanges	1679	41.57	23.36	<0.001	0.27	<0.001	1	0.88	<0.001
OVERALL	12790	46.38	27.44		0.419		2.1	1.01	

Fracture location	Number	BMI mean	BMI Standard Deviation	P-value	% Surgically Managed	P-value	CCI mean	CCI Standard Deviation	P-value
Clavicle	1236	25.32	4.60	0.376	27.61	0.008	1.71	2.40	0.013
Scapula	646	26.17	5.16	<0.001	21.06	<0.001	1.82	2.30	0.003
Proximal Humerus	1141	26.28	5.70	<0.001	18.31	<0.001	3.39	2.38	<0.001
Shaft of Humerus	361	25.95	6.52	0.361	45.39	<0.001	1.99	2.46	<0.001
Distal humerus	864	23.36	6.59	<0.001	39.8	<0.001	1.14	2.01	<0.001
Proximal Radius	437	25.91	6.14	0.015	37.27	0.007	1.41	2.03	0.245
Shaft of radius	533	22.98	7.11	<0.001	61.36	<0.001	0.30	0.92	<0.001
Distal radius	2571	25.15	6.18	0.989	35.66	<0.001	1.76	2.26	<0.001
Proximal ulna	914	25.52	6.09	0.089	39.13	<0.001	1.44	1.89	0.206
Shaft of ulna	558	23.51	7.37	<0.001	62.02	<0.001	0.40	1.10	<0.001
Distal ulna	946	24.42	6.18	<0.001	38.3	<0.001	1.41	2.19	0.093
Carpal bone(s)	381	25.83	4.59	0.038	32.02	0.739	1.17	2.12	0.002
Metacarpal(s)	523	26.01	8.24	0.003	21.8	<0.001	1.16	1.96	<0.001
Phalanges	1679	25.44	6.66	0.077	5.42	<0.001	0.97	1.63	<0.001
OVERALL	12790	25.15	6.39		31.2		1.54	2.30	

**Table 6:** Breakdown of the one-way ANOVA analysis of various demographic and management factors across each year included in the study

Factor	Comparison	Sum of Squares	Degrees of freedom	Mean Square	F value	Significance level
% surgically managed	Between Groups	2.627857676	7	0.375408239	1.51630598	0.15650343
	Within Groups	2452.535346	9906	0.247580794		
	Total	2455.163204	9913			
Age (years)	Between Groups	16560.355	7	2365.765	3.14678505	0.00253176
	Within Groups	7447368.561	9906	751.8038119		
	Total	7463928.916	9913			
Gender (% female)	Between Groups	2.174393575	7	0.310627654	1.26288553	0.26446108
	Within Groups	2436.545094	9906	0.245966595		
	Total	2438.719488	9913			
ASA grade	Between Groups	37.6111646	7	5.373023514	5.26866289	5.1789E-06
	Within Groups	5537.55636	5430	1.019807801		
	Total	5575.167525	5437			
BMI	Between Groups	354.6901431	7	50.67002045	1.23992629	0.27665226
	Within Groups	317319.4354	7765	40.86534905		
	Total	317674.1255	7772			
CCI score	Between Groups	189.7456778	7	27.1065254	5.13260074	7.6629E-06
	Within Groups	52316.0195	9906	5.281245659		
	Total	52505.76518	9913			

**Table 7:** Breakdown of the post-hoc analysis for the Bonferroni method for age, ASA grade and CCI score

Dependent Variable	(I) year	(J) year	Mean Difference (I-J)	Std. Error	Sig.	95% Confidence Interval	
						Lower Bound	Upper Bound
Age	2015	2016	-1.266	1.060	1.000	-4.579	2.047
		2017	-1.260	1.034	1.000	-4.492	1.971
		2018	-1.321	1.024	1.000	-4.520	1.878
		2019	-1.669	1.031	1.000	-4.891	1.553
		2020	-4.130*	1.113	0.006	-7.606	-0.654
		2021	-3.580*	1.089	0.028	-6.984	-0.177
		2022	-3.216	1.248	0.279	-7.116	0.683
	2016	2015	1.266	1.060	1.000	-2.047	4.579
		2017	0.006	1.062	1.000	-3.312	3.323
		2018	-0.055	1.052	1.000	-3.341	3.231
		2019	-0.403	1.059	1.000	-3.712	2.905
		2020	-2.864	1.138	0.333	-6.420	0.693
		2021	-2.314	1.115	1.000	-5.799	1.171
		2022	-1.950	1.271	1.000	-5.921	2.021
	2017	2015	1.260	1.034	1.000	-1.971	4.492
		2016	-0.006	1.062	1.000	-3.323	3.312
		2018	-0.061	1.025	1.000	-3.264	3.143
		2019	-0.409	1.033	1.000	-3.636	2.818
		2020	-2.870	1.114	0.280	-6.350	0.611
		2021	-2.320	1.091	0.936	-5.728	1.088
		2022	-1.956	1.249	1.000	-5.859	1.947
	2018	2015	1.321	1.024	1.000	-1.878	4.520
		2016	0.055	1.052	1.000	-3.231	3.341
		2017	0.061	1.025	1.000	-3.143	3.264
		2019	-0.348	1.022	1.000	-3.543	2.846
		2020	-2.809	1.104	0.308	-6.260	0.642
		2021	-2.259	1.081	1.000	-5.636	1.118
		2022	-1.895	1.241	1.000	-5.772	1.981
	2019	2015	1.669	1.031	1.000	-1.553	4.891
		2016	0.403	1.059	1.000	-2.905	3.712
		2017	0.409	1.033	1.000	-2.818	3.636
		2018	0.348	1.022	1.000	-2.846	3.543
		2020	-2.461	1.111	0.751	-5.933	1.011
		2021	-1.911	1.088	1.000	-5.310	1.488
		2022	-1.547	1.247	1.000	-5.443	2.349
	2020	2015	4.130*	1.113	0.006	0.654	7.606
		2016	2.864	1.138	0.333	-0.693	6.420
		2017	2.870	1.114	0.280	-0.611	6.350
		2018	2.809	1.104	0.308	-0.642	6.260
		2019	2.461	1.111	0.751	-1.011	5.933
		2021	0.550	1.165	1.000	-3.091	4.191
		2022	0.914	1.315	1.000	-3.195	5.022
	2021	2015	3.580*	1.089	0.028	0.177	6.984
		2016	2.314	1.115	1.000	-1.171	5.799
		2017	2.320	1.091	0.936	-1.088	5.728
		2018	2.259	1.081	1.000	-1.118	5.636
		2019	1.911	1.088	1.000	-1.488	5.310
		2020	-0.550	1.165	1.000	-4.191	3.091
		2022	0.364	1.295	1.000	-3.683	4.411
	2022	2015	3.216	1.248	0.279	-0.683	7.116
		2016	1.950	1.271	1.000	-2.021	5.921
		2017	1.956	1.249	1.000	-1.947	5.859
		2018	1.895	1.241	1.000	-1.981	5.772
		2019	1.547	1.247	1.000	-2.349	5.443
		2020	-0.914	1.315	1.000	-5.022	3.195
		2021	-0.364	1.295	1.000	-4.411	3.683

Dependent Variable	(I) year	(J) year	Mean Difference (I-J)	Std. Error	Sig.	95% Confidence Interval	
						Lower Bound	Upper Bound
ASA grade	2015	2016	-0.008	0.053	1.000	-0.173	0.157
		2017	-0.144	0.051	0.140	-0.304	0.016
		2018	-0.097	0.051	1.000	-0.256	0.063
		2019	-.17366*	0.052	0.022	-0.335	-0.012
		2020	-.21704*	0.055	0.002	-0.390	-0.044
		2021	-.24235*	0.055	0.000	-0.414	-0.071
		2022	-0.128	0.060	0.952	-0.317	0.061
	2016	2015	0.008	0.053	1.000	-0.157	0.173
		2017	-0.136	0.053	0.270	-0.301	0.028
		2018	-0.089	0.052	1.000	-0.253	0.075
		2019	-.16603*	0.053	0.049	-0.332	0.000
		2020	-.20941*	0.057	0.006	-0.386	-0.033
		2021	-.23472*	0.056	0.001	-0.410	-0.059
		2022	-0.121	0.062	1.000	-0.313	0.072
	2017	2015	0.144	0.051	0.140	-0.016	0.304
		2016	0.136	0.053	0.270	-0.028	0.301
		2018	0.047	0.051	1.000	-0.112	0.206
		2019	-0.030	0.051	1.000	-0.191	0.131
		2020	-0.073	0.055	1.000	-0.245	0.099
		2021	-0.099	0.055	1.000	-0.269	0.072
		2022	0.016	0.060	1.000	-0.173	0.204
	2018	2015	0.097	0.051	1.000	-0.063	0.256
		2016	0.089	0.052	1.000	-0.075	0.253
		2017	-0.047	0.051	1.000	-0.206	0.112
		2019	-0.077	0.051	1.000	-0.237	0.083
		2020	-0.120	0.055	0.793	-0.292	0.051
		2021	-0.146	0.054	0.208	-0.316	0.024
		2022	-0.032	0.060	1.000	-0.219	0.156
	2019	2015	.17366*	0.052	0.022	0.012	0.335
		2016	.16603*	0.053	0.049	0.000	0.332
		2017	0.030	0.051	1.000	-0.131	0.191
		2018	0.077	0.051	1.000	-0.083	0.237
		2020	-0.043	0.056	1.000	-0.217	0.130
		2021	-0.069	0.055	1.000	-0.241	0.103
		2022	0.045	0.061	1.000	-0.144	0.235
	2020	2015	.21704*	0.055	0.002	0.044	0.390
		2016	.20941*	0.057	0.006	0.033	0.386
		2017	0.073	0.055	1.000	-0.099	0.245
		2018	0.120	0.055	0.793	-0.051	0.292
		2019	0.043	0.056	1.000	-0.130	0.217
		2021	-0.025	0.058	1.000	-0.208	0.157
		2022	0.089	0.064	1.000	-0.110	0.288
	2021	2015	.24235*	0.055	0.000	0.071	0.414
		2016	.23472*	0.056	0.001	0.059	0.410
		2017	0.099	0.055	1.000	-0.072	0.269
		2018	0.146	0.054	0.208	-0.024	0.316
		2019	0.069	0.055	1.000	-0.103	0.241
		2020	0.025	0.058	1.000	-0.157	0.208
		2022	0.114	0.063	1.000	-0.084	0.312
	2022	2015	0.128	0.060	0.952	-0.061	0.317
		2016	0.121	0.062	1.000	-0.072	0.313
		2017	-0.016	0.060	1.000	-0.204	0.173
		2018	0.032	0.060	1.000	-0.156	0.219
		2019	-0.045	0.061	1.000	-0.235	0.144
		2020	-0.089	0.064	1.000	-0.288	0.110
		2021	-0.114	0.063	1.000	-0.312	0.084

Dependent Variable	(I) year	(J) year	Mean Difference (I-J)	Std. Error	Sig.	95% Confidence Interval	
						Lower Bound	Upper Bound
CCI Score	2015	2016	-0.132	0.089	1.000	-0.410	0.145
		2017	-0.057	0.087	1.000	-0.327	0.214
		2018	-0.088	0.086	1.000	-0.356	0.180
		2019	-0.232	0.086	0.201	-0.502	0.038
		2020	-.40886*	0.093	0.000	-0.700	-0.118
		2021	-.38046*	0.091	0.001	-0.666	-0.095
		2022	-0.226	0.105	0.858	-0.553	0.101
	2016	2015	0.132	0.089	1.000	-0.145	0.410
		2017	0.076	0.089	1.000	-0.203	0.354
		2018	0.044	0.088	1.000	-0.231	0.320
		2019	-0.100	0.089	1.000	-0.378	0.177
		2020	-0.277	0.095	0.105	-0.575	0.021
		2021	-0.248	0.093	0.222	-0.540	0.044
		2022	-0.094	0.107	1.000	-0.427	0.239
	2017	2015	0.057	0.087	1.000	-0.214	0.327
		2016	-0.076	0.089	1.000	-0.354	0.203
		2018	-0.031	0.086	1.000	-0.300	0.237
		2019	-0.176	0.087	1.000	-0.446	0.095
		2020	-.35222*	0.093	0.005	-0.644	-0.061
		2021	-.32382*	0.091	0.011	-0.609	-0.038
		2022	-0.170	0.105	1.000	-0.497	0.158
	2018	2015	0.088	0.086	1.000	-0.180	0.356
		2016	-0.044	0.088	1.000	-0.320	0.231
		2017	0.031	0.086	1.000	-0.237	0.300
		2019	-0.144	0.086	1.000	-0.412	0.123
		2020	-.32091*	0.093	0.015	-0.610	-0.032
		2021	-.29251*	0.091	0.035	-0.576	-0.009
		2022	-0.138	0.104	1.000	-0.463	0.187
	2019	2015	0.232	0.086	0.201	-0.038	0.502
		2016	0.100	0.089	1.000	-0.177	0.378
		2017	0.176	0.087	1.000	-0.095	0.446
		2018	0.144	0.086	1.000	-0.123	0.412
		2020	-0.176	0.093	1.000	-0.467	0.115
		2021	-0.148	0.091	1.000	-0.433	0.137
		2022	0.006	0.104	1.000	-0.320	0.333
	2020	2015	.40886*	0.093	0.000	0.118	0.700
		2016	0.277	0.095	0.105	-0.021	0.575
		2017	.35222*	0.093	0.005	0.061	0.644
		2018	.32091*	0.093	0.015	0.032	0.610
		2019	0.176	0.093	1.000	-0.115	0.467
		2021	0.028	0.098	1.000	-0.277	0.334
		2022	0.183	0.110	1.000	-0.162	0.527
	2021	2015	.38046*	0.091	0.001	0.095	0.666
		2016	0.248	0.093	0.222	-0.044	0.540
		2017	.32382*	0.091	0.011	0.038	0.609
		2018	.29251*	0.091	0.035	0.009	0.576
		2019	0.148	0.091	1.000	-0.137	0.433
		2020	-0.028	0.098	1.000	-0.334	0.277
		2022	0.154	0.109	1.000	-0.185	0.493
	2022	2015	0.226	0.105	0.858	-0.101	0.553
		2016	0.094	0.107	1.000	-0.239	0.427
		2017	0.170	0.105	1.000	-0.158	0.497
		2018	0.138	0.104	1.000	-0.187	0.463
		2019	-0.006	0.104	1.000	-0.333	0.320
		2020	-0.183	0.110	1.000	-0.527	0.162
		2021	-0.154	0.109	1.000	-0.493	0.185
